# Exploring the Elastomer Influence on the Electromechanical Performance of Stretchable Conductors

**DOI:** 10.1021/acsami.4c03080

**Published:** 2024-07-09

**Authors:** Samuel Lienemann, Ulrika Boda, Mohsen Mohammadi, Tunhe Zhou, Ioannis Petsagkourakis, Nara Kim, Klas Tybrandt

**Affiliations:** † Laboratory of Organic Electronics, Department of Science and Technology, Linköping University, 601 74 Norrköping, Sweden; ‡ Bio- and Organic Electronics Unit, RISE, Research Institutes of Sweden, 602 33 Norrköping, Sweden; § Stockholm University Brain Imaging Centre (SUBIC), Stockholm University, 106 91 Stockholm, Sweden

**Keywords:** stretchable electronics, nanowires, elastomers, composites, stretchable conductors

## Abstract

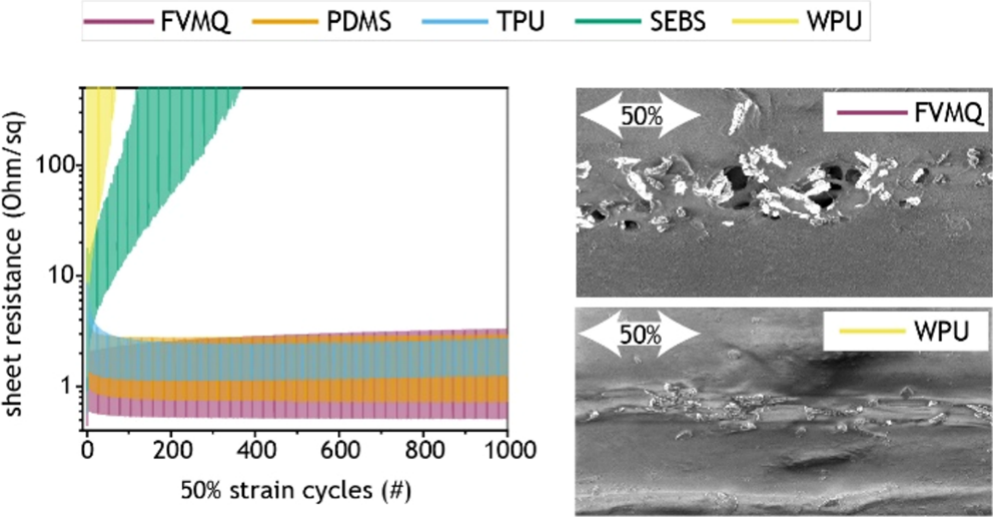

Stretchable electronics has received major attention in recent years due to the prospects of integrating electronics onto and into the human body. While many studies investigate how different conductive fillers perform in stretchable composites, the effect of different elastomers on composite performance, and the related fundamental understanding of what is causing the performance differences, is poorly understood. Here, we perform a systematic investigation of the elastomer influence on the electromechanical performance of gold nanowire-based stretchable conductors based on five chemically different elastomers of similar Young’s modulus. The choice of elastomer has a huge impact on the electromechanical performance of the conductors under cyclic strain, as some composites perform well, while others fail rapidly at 100% strain cycling. The lack of macroscopic crack formation in the failing composites indicates that the key aspect for good electromechanical performance is not homogeneous films on the macroscale but rather beneficial interactions on the nanoscale. Based on the comprehensive characterization, we propose a failure mechanism related to the mechanical properties of the elastomers. By improving our understanding of elastomer influence on the mechanisms of electrical failure, we can move toward rational material design, which could greatly benefit the field of stretchable electronics.

## Introduction

Stretchable electronics has received major attention in recent years due to the prospects of integrating electronics in clothing,^1,2^ on skin,^3−7^ and in soft implants.^8−14^ One of the major approaches for realizing stretchable electronics is through the development of stretchable conducting composites, in which a (semi)conducting filler is incorporated in an elastomer, yielding a conducting rubber-like material.^15−17^ The requirements of the composites are determined by the application, e.g., on the skin, strain sensors require in the order of 10^4^ strain cycles to 10% strain.^18^ In general, it is clear that most applications require the combination of repeatedly reversible elastic deformations and preserved conductivity, which is in line with Lipomi’s definition of stretchable electronics.^16^ A wide range of nanofillers have been investigated for stretchable conductors, including carbon black,^19^ carbon nanotubes,^2,10,12,20^ graphene,^21^ metal nanoparticles,^22,23^ metal nanoflakes,^24−26^ and metal nanowires (NWs).^14,27−34^ Some of the most commonly employed elastomers for these applications are silicones, polyurethanes, styrene–butadiene–ethylene–styrene, styrene–isoprene–styrene, fluoro-silicones, and -hydrocarbons.^15,34−39^ The electromechanical performance of a specific composite can depend on a wide range of factors, including filler properties, filler concentration, filler dispersion, filler–filler interactions, filler–elastomer interactions, and elastomer properties, altogether making up a very complex system. Indeed, the reported electromechanical performance varies a lot between composites, depending on both the compositions and fabrication methods, as highlighted in several recent review papers on the topic.^15,35−39^ Many of the best-performing composites are based on metal nanowires due to their good conductivity and high aspect ratio. Examples include silver NWs,^30,31,33,34^ copper NWs,^29^ and gold-coated NWs,^14,27,28^ warranting special interest in this class of materials. In contrast to the large body of work on the development and characterization of different stretchable conductors, the number of studies exploring the mechanisms of conductors under strain are far fewer. Carbon black received early attention both as a reinforcing agent and as a conductivity enhancer for mainly natural rubbers, butyl rubbers, and styrene–butadiene rubbers.^40−44^ Although some basic concepts regarding mechanical and electrical influence can be adopted from these studies, the electromechanical behavior of carbon black rubbers (aggregate alignment can improve conductivity with strain^45^) differs significantly from the behavior of recent highly conductive composites (decrease in conductivity with strain). Significant research has also gone into experimental and theoretical studies of the percolation threshold of conducting composites;^37^ however, high-performance conductors operate at far higher filler concentrations. For such conductors, it can be more relevant to study crack formation and propagation within the composites to understand the resistance behavior.^46,47^ A study on thick silver NWs in PDMS recorded the movement of NWs within the elastomer matrix and could in combination with numerical modeling predict the single-stretch performance of the composite.^48^

The vast majority of experimental and theoretical reports study composites comprising one or several types of fillers,^34^ different filler concentrations, aspect ratios,^32,49^ often surfactants/additives,^50^ but only one type of elastomer. This is somewhat surprising, as we have noticed big differences in the electromechanical performance of composites with the same filler but different elastomers. This seems to be true in particular for strain cycling performance, where we have noticed that some composites fail very quickly. Indeed, there are examples in the literature where highly conducting NW-based composites survive strain cycling while others fail, despite similarities in NW fillers.^15,38^ Since these studies come from different reports, it is, however, not clear what is causing these observed differences, as filler materials, coatings, and fabrication procedures could differ. It is also not known what the failure mechanisms are for the various strain-cycled composites and how that relates to elastomer properties. To address these unknowns, here we report on a systematic study of the elastomer influence on stretchable conductor performance. We fabricate samples of gold NW (AuNW) conductive networks embedded in five different elastomers of similar moduli at 100% strain but with different chemical compositions and viscoelastic behavior. The samples are thoroughly characterized by electromechanical testing, mechanical testing, surface measurements, and microscopy. Interestingly, we find that some samples fail electromechanically even though the NW film looks intact. Based on the characterizations, we propose a failure mechanism on the nano/microscale related to the elastomer behavior. The study indicates what constitutes a good elastomer for stretchable electronics and provides a starting point for further studies in the failure mechanisms of stretchable conductors.

## Results

### Sample Composition and Fabrication

**Figure 1:**
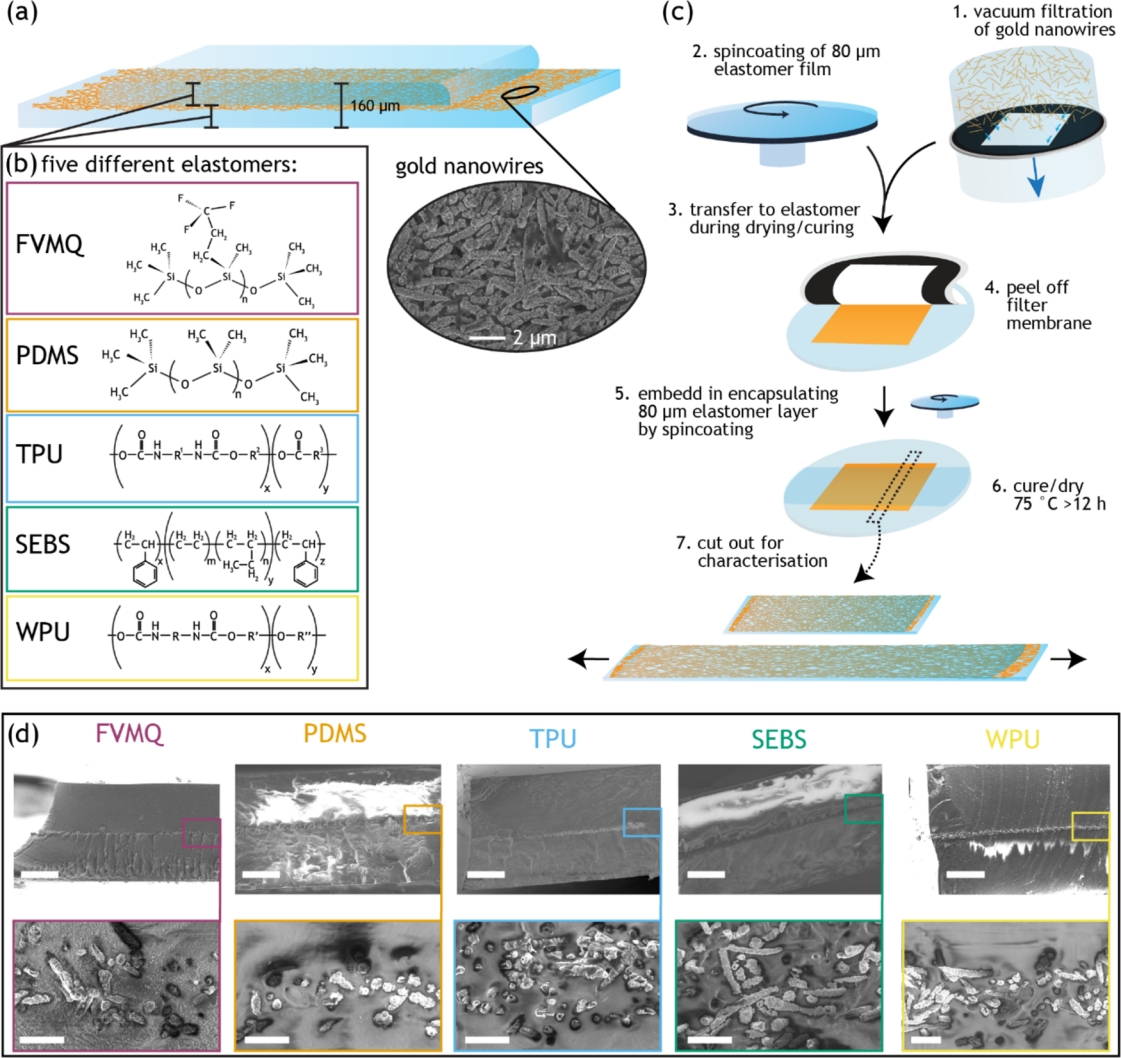
Stretchable conductors based on five elastomers. (a) Schematic of the sample with gold nanowire (AuNW) film embedded in five different elastomers with the SEM image of the AuNW embedded in PDMS at the open contact. (b) Generalized chemical structure of the five elastomers and their color code in this manuscript (see also Table 1). FVMQ, purple; PDMS, orange; TPU, blue; SEBS, green; WPU, yellow. (c) General fabrication process for all of the samples. Detailed parameters for the different elastomers can be found in the Supporting Information. (d) SEM cross-sectional images showing the AuNWs embedded in the five elastomers. Scalebars: top 50 μm, bottom 2 μm.

Five elastomers of different chemical compositions but of similar moduli at 100% strain were chosen for the study (Table 1 and Figure 1b). The elastomers were chosen to represent common material classes, with one poly(dimethylsiloxane) (PDMS), one fluoro-silicone (FVMQ), one styrene-based block copolymer (SEBS), and two polyurethanes (TPU and WPU) of which one comes as water-dispersed nanoparticles. Two of the elastomers are thermosets with reinforcing silica particles (Nusil MED10-5440, Dow Sylgard 184 (PDMS)), while the other three are thermoplastics (Elastollan Soft 35 A 12 (TPU), Tuftec H1052 (SEBS), Alberdingk U 4101 VP (WPU)).

**Table 1:**
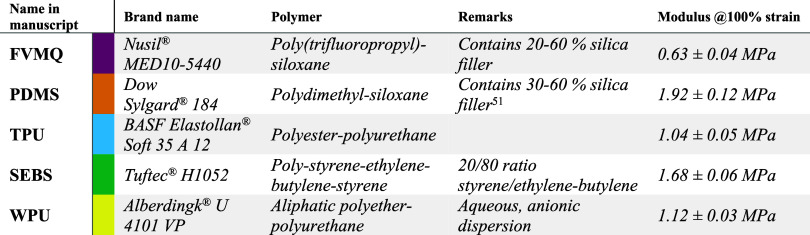
Elastomers Used in This Study, Including Brand Name, Composition, and Measured Modulus at 100% Strain

A standard test sample structure was developed by embedding an ∼5 μm thick (Figure S1) film of AuNWs in between two 80 μm thick layers of elastomer to facilitate direct comparison of performance (Figure 1a). Encapsulated stretchable conductors are common in stretchable circuit boards, and here, the conductive layer was placed in the neutral bending plane to minimize the effect of bending during peeling and sample handling. A filtration and transfer method was used to deposit the AuNW film on top of the elastomers, resulting in a similar AuNW film density (∼2.7 mg/cm^2^) and thickness (Figure S1) for the various elastomer samples, with an approximate filler volume fraction of 28%. A second elastomer layer was coated on top of the transferred AuNWs to infiltrate and encapsulate the NW network (Figure 1c). The transfers of the AuNWs to the thermosets were facilitated by semicured elastomer films, while the transfers to the thermoplastics were enabled by the remaining solvents in the elastomer films. All elastomers infiltrated the AuNW layer during sample fabrication (Figure 1d).

### Electromechanical Performance

**Figure 2:**
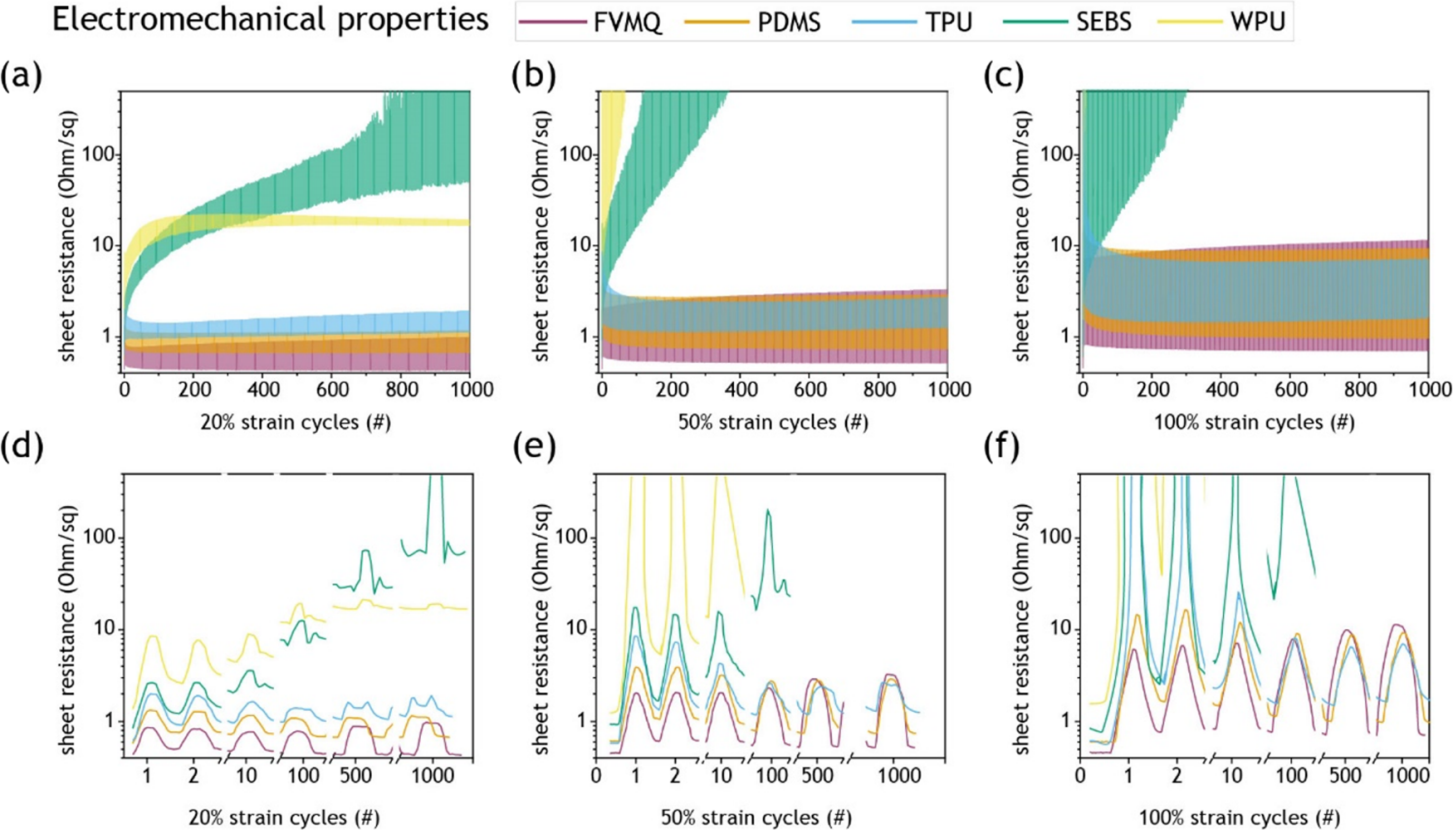
Sheet resistance-strain measurements for AuNW stretchable conductors based on different elastomeric matrices: FVMQ (purple); PDMS (orange); TPU (blue); SEBS (green); and WPU (yellow). (a–f) Mean sheet resistance (*n* = 3) during 1000 stretch cycles to (a, d) 20%, (b, e) 50%, and (c, f) 100% strain. (a–c) Full cycle range and (d–f) specific cycles.

**Figure 3:**
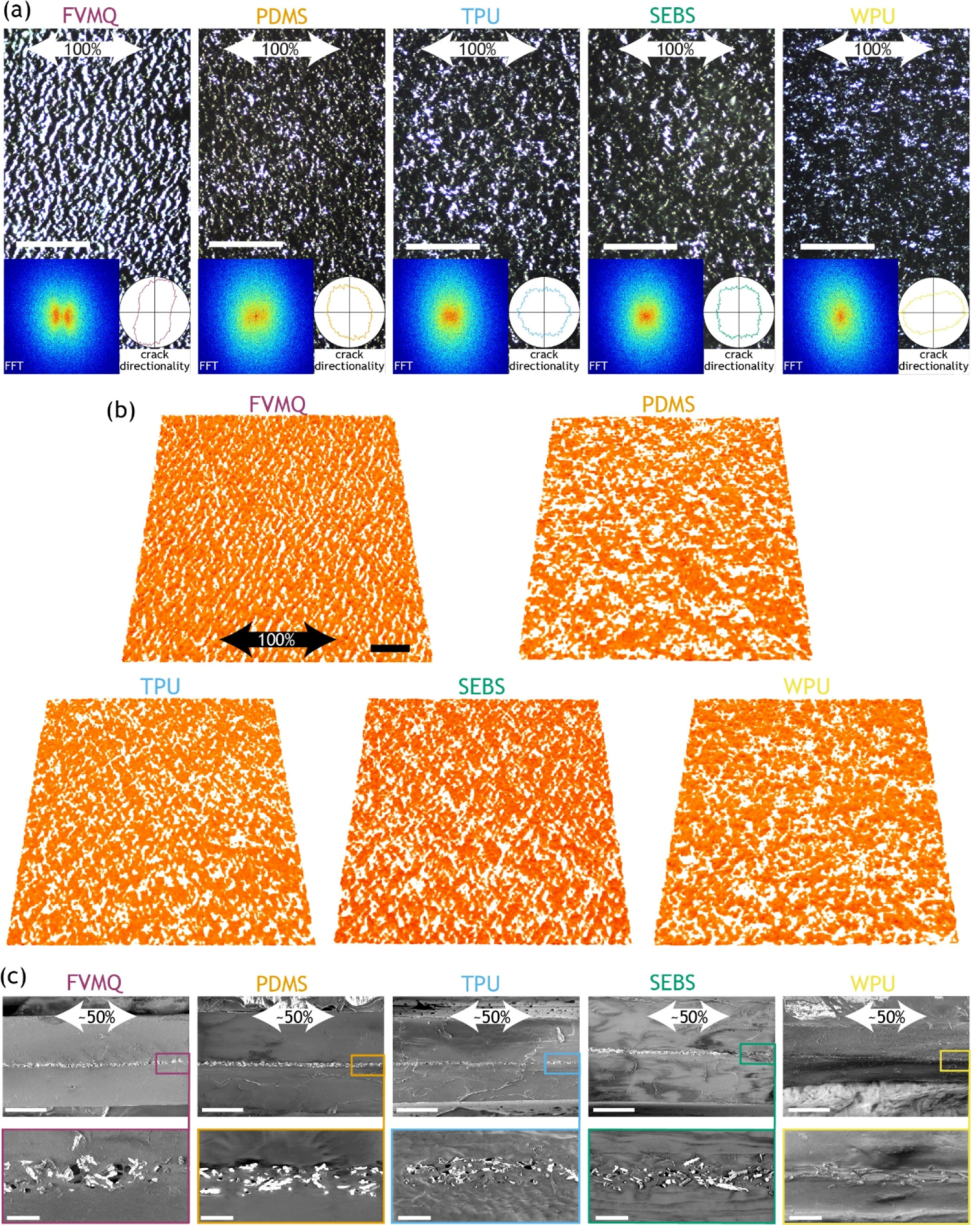
Structural characterization of strain-cycled AuNW films. (a) Representative backlight top view microscope pictures after 1000 cycles to 100% strain, taken at 100% strain. The insets show the 2D-fast Fourier transform (FFT) of the images highlighting the crack periodicity (left inset) and the crack directionality (right inset). Scalebars: 100 μm. (b) X-ray tomography of AuNW films after 1000 cycles to 100% strain, taken at 100% strain. The elastomer substrate is not visible, only a square of the ∼5 μm thick AuNW films, which are viewed at a tilted angle. Scalebar: 50 μm. (c) Longitudinal cross-sectional SEM images of 100% strain-cycled samples at ∼50% strain. Scalebars: top 50 μm, bottom 5 μm.

The electromechanical performance was evaluated by strain cycling the elastomer–AuNW samples to 20, 50, and 100% strain for 1000 cycles (Figure 2a–f). The measurements were performed in a customized linear stretching setup with four-probe contacts to avoid contact resistance contributions. The samples were taped and clamped slightly into the insulated part to avoid localized damage at the more sensitive contact pads (Figure S2). At 20% strain, the FVMQ, PDMS, and TPU performed well, while the SEBS and WPU already showed deteriorating performance (Figure 2a,d). At 50% strain, the FVMQ, PDMS, and TPU still showed stable performance, while the SEBS and WPU samples lost conductivity fast (Figure 2b,e). The same trend was present at 100% strain, with the WPU becoming nonconductive after a few cycles and the SEBS gradually losing conductivity over hundreds of cycles (Figure 2c,f). The FVMQ samples generally had the lowest resistance in the relaxed state but slightly higher resistance than the PDMS and TPU at max strain. The TPU showed a strong increase in resistance during the first two cycles but recovered to have the lowest change in resistance between stretched and relaxed states throughout the measurements. The backlight microscopy images of the cycled samples at 100% strain are shown in Figure 3a, while the images in the relaxed state and after 20 and 50% cycling can be found in Figure S3. The FVMQ sample exhibited clear cracks in the AuNW layer perpendicular to the strain direction, while the PDMS sample had smaller cracks in the same direction. The TPU and SEBS samples showed rather randomly oriented cracks, while the cracks of the WPU tended to be oriented in the stretching direction. The tendency in crack periodicity and directionality was confirmed by the 2-dimensional fast Fourier transform (2D FFT) maps and crack directionality shown as insets for the various backlight images, with only FVMQ showing a crack periodicity of ∼9 μm in the strain direction. Overall, there were no long-range cracks visible that could cause full breaks in the conductors, with the FVMQ exhibiting the largest cracks of up to ∼50 μm in length. The structure of the AuNW layers was further studied by X-ray tomography (Figures 3b and S10). The 3D reconstructions revealed that all AuNW films were rather planar during stretching. Again, the crack structure in the FVMQ was more defined and directional in comparison with the other samples, among which no distinct differences could be observed. SEM images in Figure 3c reveal void formation between the elastomer and AuNWs during strain, that is, the NWs partially detach from the elastomer in the strain direction for all but the WPU composite. For WPU, a smearing out of elastomer and realignment of the AuNWs in the strain direction are visible instead.

### Elastomer–Gold Interface Characterization

**Figure 4:**
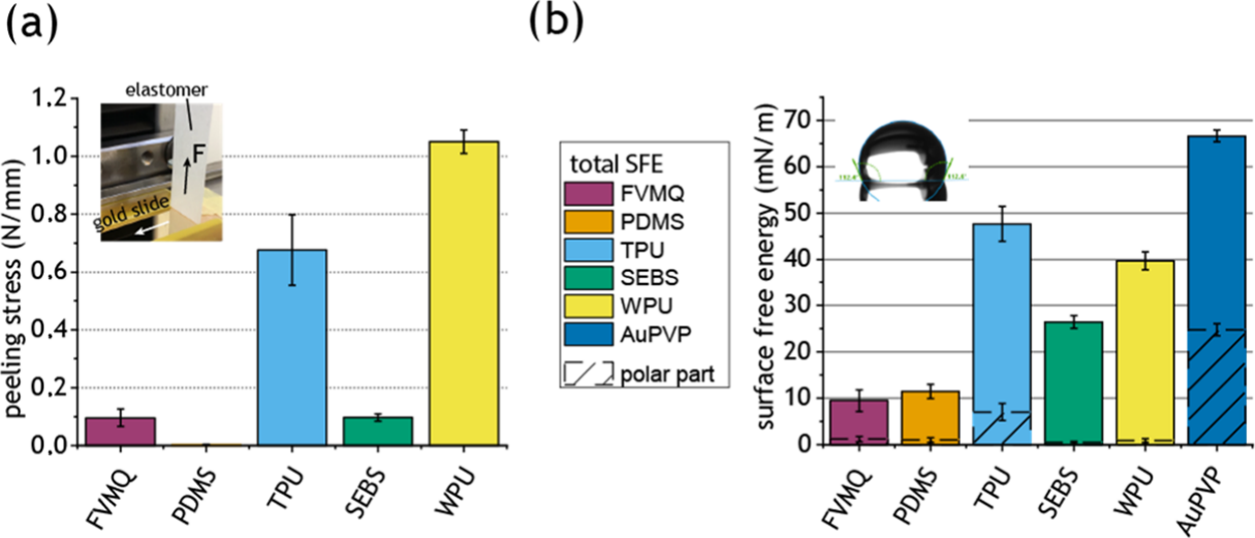
Interface properties. (a) Peeling stress needed to peel elastomer samples from poly(vinylpyrrolidone)-treated gold slide (AuPVP) in a 90° peel test (*n* = 3, with standard deviation). (b) Total surface free energy (SFE, dispersive and polar) of interfaces after peeling. The hashed area denotes the polar contribution (*n* = 3, with standard deviation). (a, b) Insets show images of the measurement setup.

The adhesion between the filler and elastomer has been reported to influence the properties of the composite.^50^ To evaluate the adhesion between the five elastomers and gold, 90° peel tests were performed. Gold slides were first incubated in poly(vinylpyrrolidone) (PVP) solution and then rinsed to resemble the processing of the AuNWs. The elastomer films were then casted on the gold slides (AuPVP), reinforced, and peeled. The mean peeling force was the lowest for PDMS (∼0 N/mm), followed by FVMQ (0.09 N/mm) and SEBS (0.10 N/mm); see Figure 4a. TPU exhibited a significantly higher mean peeling force of 0.68 N/mm, only topped by 1.05 N/mm for WPU (Figure 4a). TPU and WPU were hard to peel, with TPU occasionally leaving small traces of material on the gold slide after peeling. One of the factors affecting the adhesion is the surface free energy (SFE) of the interfacing surfaces,^50^ which was measured for both the AuPVP surface and the surface of the peeled elastomers. The AuPVP surface had a high SFE of 66.7 mN m^–1^, with a substantial polar part. FVMQ and PDMS showed the lowest SFE averages at 9.5 ± 2.4 and 11.5 ± 1.5 mN m^–1^, respectively, which correlates well with low adhesion to the AuPVP surface. The WPU and TPU had the highest SFEs of the elastomers at 39.6 ± 1.9 and 47.7 ± 3.8 mN m^–1^, respectively, which also correlates well with high adhesion to AuPVP. The SEBS had a medium SFE (26.5 ± 1.3 mN m^–1^) but low adhesion to the AuPVP. All elastomers exhibit similar SFE (polar and dispersive) for the surface on top and the one in contact with the AuPVP after peeling except TPU, which had a much higher SFE at the interface to AuPVP; see Figure S4a. It should be noted that the AuPVP surface after peeling of the elastomers showed a slight reduction in SFE (both dispersive and polar parts); see Figure S4b.

### Mechanical Characterization

**Figure 5:**
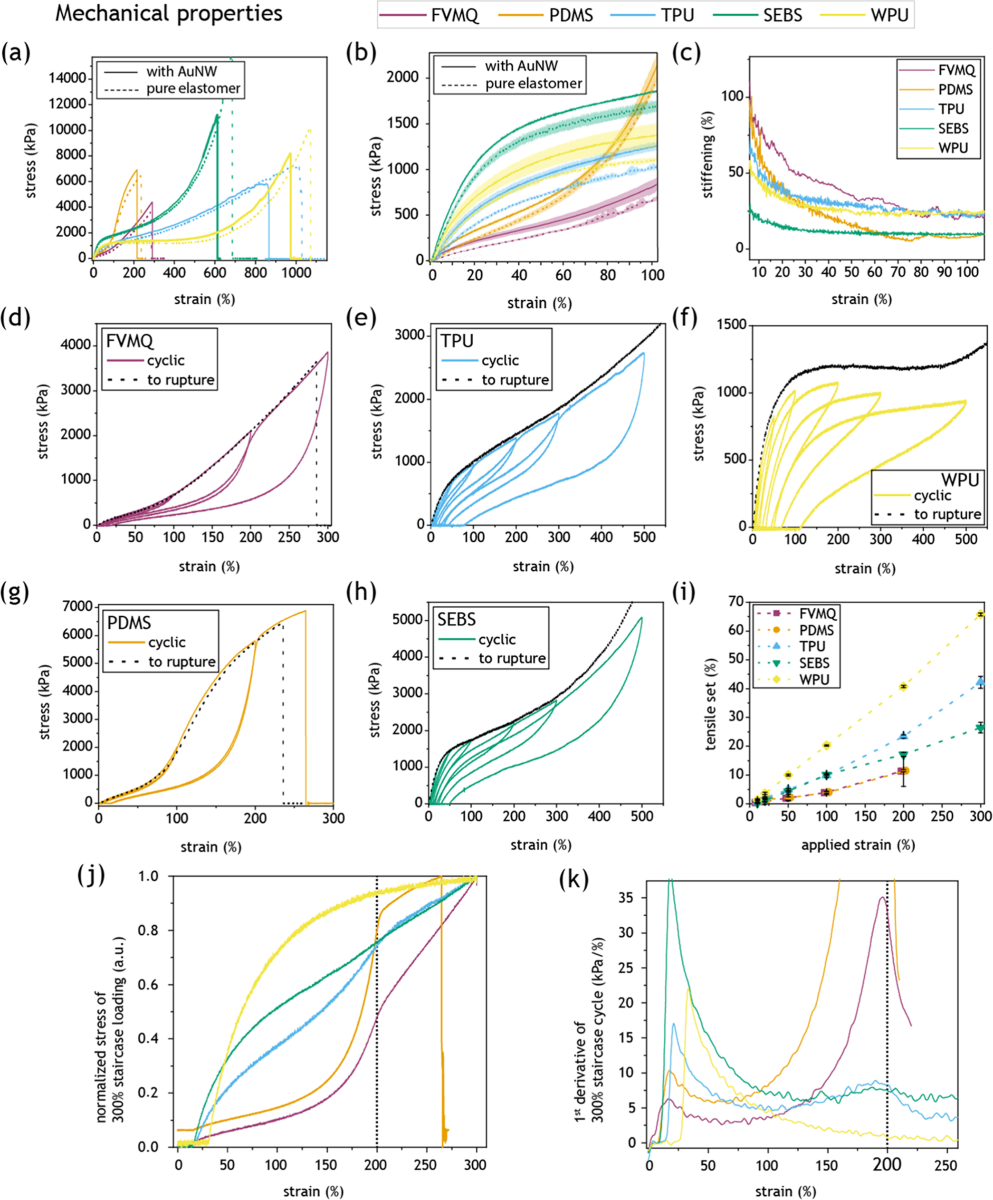
Mechanical properties of the five elastomers. FVMQ, purple; PDMS, orange; TPU, blue; SEBS, green; and WPU, yellow. (a) Stress–strain curves until rupture of representative elastomer samples with (solid line) and without (dotted line) AuNWs. (b) Stress–strain curves at low strains with mean and standard deviation (shaded area) of elastomer samples (*n* = 3) with (solid line) and without (dotted line) AuNWs. (c) Stiffening of the different elastomer samples due to the AuNW layer. (d–h) Representative stress–strain curves for cyclic loading of pure elastomers, during incrementally increasing strain to 10, 20, 50, 100, 200, 300, and 500% (solid, colored line) with the representative tensile loading curve to rupture (dotted, black line) for (d) FVMQ, (e) TPU, (f) WPU, (g) PDMS, and (h) SEBS. (i) Tensile set for pure elastomer during cyclic loading (mean and standard deviation, *n* = 3). (j) Representative stress–strain curve to 300% of pure elastomers after a previous cycle of 200% strain. (k) 1st derivative of mean stress values up to 300% strain after a previous cycle of 200% strain (see (j)). (j, k) The previously applied strain of 200% is marked with a dotted line at the *x*-axis.

The mechanical properties of the five elastomers were characterized to investigate the relationship between mechanical and electromechanical properties. The stress–strain curves until rupture for the elastomers with and without AuNWs are shown in Figure 5a,b. All of the elastomers had an average modulus in the range of 0.6–1.9 MPa at 100% strain. The thermosets (FVMQ and PDMS) had the lowest fracture strain at 200–300%, while the thermoplastics showed significantly higher fracture strains at 600–1000%. The thermosets had a relatively low Young’s modulus (slope of the stress–strain curve; see Figure S5) for low strains and sharp strain stiffening for elevated strains. In contrast, the thermoplastics initially had high moduli, which decreased significantly for strains >50%, until a final strain stiffening phase before rupture (except for TPU). The relative stiffening of the samples by the AuNW network was generally higher at low strains and gradually decreased for higher strains (Figure 5c). For low strain (<10%), FVMQ and PDMS experienced the most stiffening, followed by TPU and WPU, with SEBS standing out with only a little initial stiffening.

Figure 5d–h shows the stress–strain curves for cyclic strain applied with increasing amplitude to the elastomers. The impact of the previous strain cycles differs significantly between the elastomers. FVMQ and PDMS show minor hysteresis in forward and backward scans up to 100% strain but major hysteresis for 200% and above. SEBS had moderate hysteresis at all strains, TPU a bit more, and WPU by far the most hysteresis of the elastomers. The relative remaining elongation upon release of the stress is called the tensile set, which was (for 100% strain) around 4% for FVMQ and PDMS, 10% for TPU and SEBS, and 20% for WPU (Figure 5i). The set follows approximately the same trend in between the elastomers as the hysteresis in the stress–strain curves. Another interesting mechanical aspect that differs between the elastomers is the observed strain softening from previous strain cycles. FVMQ, PDMS, and TPU approximately follow the previous relaxation curve in the next forward scan up until the point of the previous max strain, at which they approximately join the curves of previously unstrained samples (Figure 5d,e,g). This behavior is indicative of nonrecoverable broken bonds.^43^ SEBS and WPU experienced a general softening from previous strain cycles, as they followed the overall lower loading curves for the full strain range (Figure 5f,h), indicating partial reformation of bonds. The difference in behavior is highlighted in Figure 5j, where the curves for SEBS and WPU remained unchanged when scanning past the previous max strain, while FVMQ, PDMS, and TPU showed distinct changes in their curves around the previous max strain. The differences around the previous max strain become even more evident by looking at the derivative of the stress–strain curves (Figure 5k).

**Figure 6:**
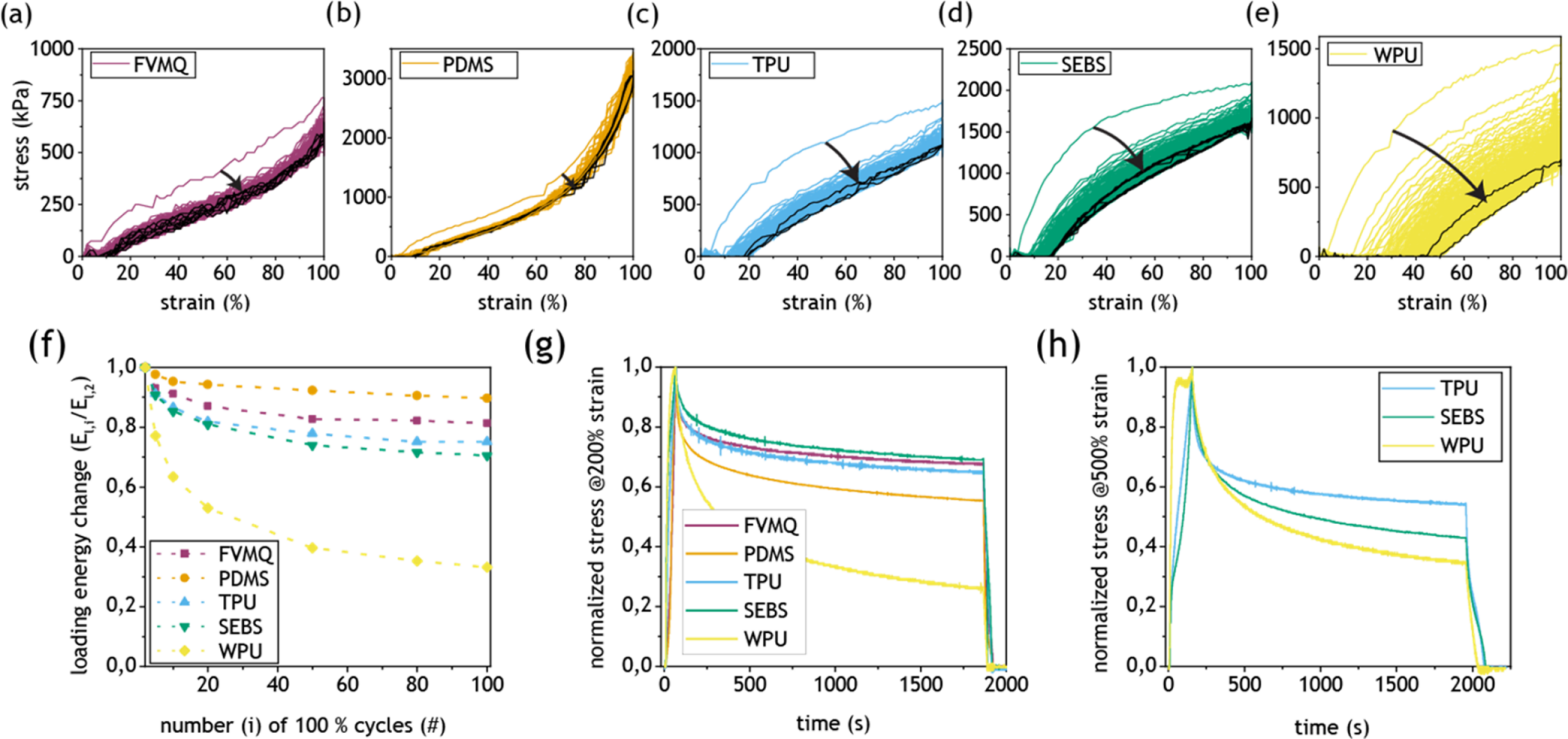
Time-dependent mechanical behavior. (a–e) Stress–strain curves of elastomer–AuNW samples during 100 cycles to 100% strain. The arrows indicate changes from the first to the 100th cycle. (f) Change in the loading energy (area below the curve) during consecutive strain cycling to 100% strain. Energy of each loading cycle (*E*_l,i_) is divided by the energy during the second loading cycle (*E*_l,2_) to highlight the trend during later cycles. (g, h) Stress relaxation normalized to highest stress during 30 min for pure elastomer samples (g) at 200% strain and (h) at 500% strain (PDMS and FVMQ rupture at lower strains and are not included).

The mechanical effect of strain cycling can be of importance for understanding the electromechanical performance of the samples. Figure 6a–e shows the evolution of the stress–strain curves during 100 cycles of elastomer–AuNW samples to 100% strain. PDMS showed the most reversible behavior, followed by FVMQ. TPU and SEBS exhibit similar hysteresis, with SEBS having a slightly more gradual change throughout the cycles. WPU showed the largest hysteresis by far and only returned to 50% strain upon relaxation after 100 cycles. The extracted energy during several loading cycles for the different elastomers is shown in Figure 6f. Stress relaxation measurement is another way of characterizing time-dependent changes in elastomers. Figure 6g,h shows stress relaxation measurements for the elastomers at 200 and 500% strain. WPU experienced the strongest stress relaxation at both strains, while FVMQ, TPU, and SEBS showed similar behavior at 200% strain. At 500% strain, SEBS had stronger relaxation than TPU.

## Discussion

The aim of this work was to investigate the effect of different elastomers on the electromechanical performance of stretchable conductors. The sample preparation method was highly suitable for this task, as the AuNW layers were formed separately and then transferred onto the elastomers, thereby avoiding dispersion of the filler in different systems, which could have rendered structural differences in the composites. Indeed, SEM images confirmed successful embedding of the AuNWs in the various elastomer substrates (Figure 1d), and the initial sheet resistance was similar for the different elastomers (Figure S6), further indicating good quality of the formed conductors, with only the sheet resistance of the WPU samples showing significantly higher initial values once mounted in the measurement setup (Figure S7), as the minor strain during peeling already exposed the suboptimal electromechanical properties of the composite.

We chose sheet resistance during strain cycling as the figure of merit for our stretchable conductors based on the conductor requirements in relevant applications. During the first cycles at 20% strain, all elastomer samples perform reasonably; however, the WPU and SEBS sample performances deteriorated rapidly with additional cycles (Figure 2a,d). This demonstrates why single strain tests are insufficient for characterizing stretchable conductor performance and why strain cycling is necessary. Cycling to higher strain increased the rate of performance deterioration for the WPU and SEBS samples, while the FVMQ, PDMS, and TPU samples all exhibited stable performance even at 100% strain cycling (Figure 2c,f). Interestingly, for higher strains, the TPU samples showed a sharp resistance increase during the first cycle, after which the resistance gradually recovered back down and stabilized. This behavior is the opposite of that of WPU and SEBS, although similar behavior of stretchable conductors has previously been reported.^52,53^ The strong effect of strain cycling becomes even more evident while comparing strain cycling to single strain performance (Figure S8). For lower single strains (e.g., 20%), TPU and SEBS perform similarly (Figure S8), while for strain cycling, SEBS perform much worse than TPU. The strain rate had no major effect on the relative performance of the different conductors (Figure S9). Interestingly, backlight microscopy and X-ray tomography revealed no macroscopic cracks in the cycled WPU and SEBS samples and the AuNW films looked rather well interconnected despite their loss of conductivity (Figures 3a,b and S3). Instead, the conductive FVMQ sample revealed major crack formation perpendicular to the strain direction, and denser smaller cracks were also visible in the PDMS sample. This demonstrates that macroscopic cracks can be consistent with good electromechanical performance, although there seems to be a trend that larger cracks generate a larger increase in resistance upon strain. In contrast, the absence of macroscopic cracks can be consistent with electrical failure in the conductors. This indicates that the failure mechanism is not related to the macroscopic scale but rather to micro/nanoscopic rearrangements within the conductors upon strain.

When evaluating possible micro/nanoscopic failure mechanism for the conductors, one needs to consider the rearrangements around the NW network upon strain. The NW network restricts elastomer deformation and causes local high-stress regions, which can lead to microscopic ruptures at the elastomer–NW interface during stretching. While such ruptures are apparent for FVMQ, PDMS, TPU, and SEBS (Figure 3c), the stretched WPU instead shows reformation of the elastomer matrix around the NW network and realignment of AuNWs in the strain direction. The fact that stiffening is observed for all of the AuNW composites (Figure 5c) further indicates that local high-stress regions are formed around the network, where deformations are restricted by the NWs. As interactions between the filler and the elastomer matrix are crucial to the micro/nanoscopic stress response and known to affect the properties of the composite,^43,50^ next we investigated the adhesion and surface properties of the different elastomers. The FVMQ, PDMS, and SEBS surfaces had low adhesion to the AuPVP surface, while TPU and WPU had high adhesion (Figure 4a). The measured surface free energy was consistent with this trend, as the TPU, WPU, and AuPVP surfaces had the highest surface free energy (Figure 4b). When looking at the stiffening effect of the AuNW film on the samples, however, FVMQ and PDMS exhibited the highest relative stiffening, followed by TPU and WPU. This is inconsistent with the low adhesive forces for FVMQ and PDMS but consistent with the higher adhesion of TPU and WPU. Also, SEBS showed low stiffening, in line with its low adhesion. Possible explanations for the unexpected behavior of the FVMQ and PDMS samples could be their strain stiffening characteristics, which enhance the stiffening contribution from local high strain regions. Another explanation could be that the inherently high loading of silica nanoparticles within the FVMQ and PDMS restricts local deformations within the AuNW network even further. Regardless, the trend in stiffening cannot explain the observed variation in electromechanical performance between the studied samples, as TPU and WPU experienced a similar degree of stiffening but had very different electromechanical performance.

The investigation continued with mechanical characterization of the samples by the application of cyclic strain of incrementally increasing magnitude (Figure 5d–h). The thermoset (FVMQ, PDMS) samples had similarly shaped stress–strain curves, while the thermoplastics’ (TPU, SEBS, WPU) stress–strain curves had their similarities, although WPU showed more plastic deformations. This is not surprising, as the thermosets are both silicones with high loading of covalently cross-linked silica particles, giving their polymer network a certain characteristic.^51^ In contrast, the thermoplastics are physically cross-linked by immobile polymer domains, giving rise to a different polymer network. This difference is also manifested in the lower tensile set of the thermosets, indicating that the structure of the polymer network is more preserved during strain for them than for the thermoplastics. So far, the mechanical behavior of the TPU and SEBS has been rather similar, providing no hints on what could be responsible for their vastly different electromechanical performance. However, one aspect that differentiates the two elastomers is how strain softening is manifested in the materials (Figure 5d–k). The strain softening in the thermosets had a clear memory effect of the previously reached maximum strain, as the strained samples were softer up until the previously reached strain, after which the softening effect disappeared. This behavior, often called the Mullins effect, is indicative of the irreversible breaking of bonds, common during the first deformation of filled or crystallizing elastomers.^43,44^ The SEBS and WPU became generally softer from previous strain but showed no sharp transition at the previously reached maximum strain (Figure 5j,k). This is indicative of a general rearrangement and reformation of physical cross-links within the network, leading to softening even above the previously reached maximum strain. Indeed, atomic force microscopy studies^54^ and high-resolution SEM imaging^34^ have shown that the cross-linking domains within SEBS reform due to applied strain. The strain softening of TPU is interesting in this context, as it showed a mixed response with both general softening and a memory effect. This difference in response to strain between TPU and SEBS could manifest itself during cycling experiments, where materials with permanently broken cross-links would be expected to change most during the first strain cycle, while materials with reforming bonds would be expected to have a more continuous change. Indeed, strain cycling measurements of the elastomer–AuNW samples showed that the thermosets had less continuous change, while the thermoplastics had a more gradual change, especially in the case of WPU (Figure 6a–f). The TPU had slightly less continuous change than the SEBS, indicated by the shallower slope of the change in loading energy in Figure 6f, which also showed up in stress relaxation measurements at 500% strain (Figure 6h).

To summarize, we have observed large variations in electromechanical performance between the different elastomer–AuNW samples, with FVMQ, PDMS, and TPU performing well and SEBS and WPU performing poorly. Backlight microscopy images and X-ray tomography revealed no macroscopic cracks in the SEBS and WPU samples, indicating that the failure mechanism is on the nano/microscale in the AuNW network. The elastomers had large variations in adhesion to AuPVP surfaces, but the trend was inconsistent with the electromechanical performance. Differences in stiffening by the AuNW network could neither account for the variations in the electromechanical performance of the samples. The overall shape of the stress–strain curves showed many similarities between the TPU and SEBS samples; however, there were some variations in how the materials reacted to strain, with SEBS showing general strain softening, while TPU had some memory effect of previous strain, similar to the thermosets. Further indications of differences in the reaction to strain were found for strain cycling and strain relaxation measurements.

Finally, based on the above observations and reasoning, we propose a failure mechanism for the SEBS and WPU conductors. We believe that the main mechanism for the loss in conductivity is the lasting displacement of AuNWs due to high local strains within the elastomer matrix, making them lose their electrical connections to neighboring AuNWs. One should note that the local strain levels around the AuNWs can be much higher than the macroscopic sample strain, as the NWs restrict the deformations within the composite. Thus, it is likely that small fractures or creeps occur within the elastomer around the NWs. Only a few nanometers of material in between adjacent AuNWs would be sufficient to drastically decrease the conductivity of the network. Lasting displacements of NWs would be facilitated by reformation of the elastomer network, which was found not only to a higher degree in the WPU (worst performance) but also to some extent in SEBS (poor performance). This would also explain why the same failure was not observed in the FVMQ-, PDMS-, and TPU-based conductors, as those elastomers had more fracturing of cross-links rather than reformation of cross-linking domains. The ability of SEBS and WPU samples to slowly recover from nonconductive to moderately conductive after strain cycling (Figure S11) further indicates that reformulations of the polymer networks through viscoelastic effects and cross-link remodulation are crucial for the performance.

## Conclusions

We have fabricated and characterized stretchable conductors based on AuNWs and five chemically different elastomers: two thermosets and three thermoplastics. The choice of elastomer had a huge impact on the electromechanical performance of the conductors under cyclic strain, as FVMQ, PDMS, and TPU performed well, while SEBS and WPU failed rapidly at 100% strain cycling. Microscopy revealed no major cracks in the electrically failing composites, indicating that the failure occurred on the micro/nanoscopic level. TPU and SEBS had overall similar mechanical characteristics; however, the strain softening response in the elastomers was different, pointing toward the fact that SEBS reformed the cross-linking network under strain. We therefore suggest that the electrical failure in SEBS and WPU originates from lasting reformulations of the viscoelastic elastomer matrices and displacements of the AuNWs within the network during strain cycling, which together degrades the electrical connectivity between the AuNWs. The huge difference in performance, in combination with visually intact conducting layers, is a very interesting observation with respect to stretchable composite conductors. It indicates that the key aspect for good electromechanical performance is not homogeneous films on the macroscale but rather beneficial interactions and changes on the nanoscale. As the exact mechanism of these nanoscale changes, and what elastomer properties that drive them are largely unknown, this is an attractive topic for future studies. The generality of the finding can be assessed by studying more combinations of elastomers and fillers. One should note that in this work stretchable composites that are encapsulated on both sides are studied. The thickness of the encapsulation layer can affect the electromechanical performance, although the effect is often rather small (see AuNW–PDMS with 40^27^ and 10 μm^28^ encapsulations). Samples with no or one-sided encapsulation might perform worse, however.^55^ Sophisticated imaging techniques, including X-ray diffraction techniques, could help resolve the changes in the conductive network and thereby provide additional details on the involved processes. We believe this study is of importance to the field of stretchable electronics, as it shows the huge impact of elastomer choice when designing stretchable composites. Much of the previous work has been focused on optimizing fillers for one elastomer system, when in fact the performance of stretchable composite electronics can depend largely on the choice of elastomer rather than the filler. This study also highlights the need for a better understanding of the mechanisms that determine a good or bad stretchable conductor. By improving our knowledge about the elastomer choice and the mechanisms of electrical failure, we can move toward rational material design, which will greatly benefit the field of stretchable electronics.

## Experimental Section

### Stretchable Conductors

#### Gold Nanowire Network

Gold nanowires (AuNWs, ∼350 nm diameter, 5–10 μm length) were fabricated in-house via a previously reported gold reduction process in solution.^28^ 20 mL of AuNW solution (≈0.85 mg/mL) was vacuum-filtered onto a PVDF filter membrane (Merck Millipore HVLP04700, 0.45 μm pore size) with a 625 mm^2^ square pattern on top to a resulting film thickness of ∼5 μm. Subsequently, the deposited AuNW network was cleaned from residual poly(vinylpyrrolidone) and other synthesis residues by filtering an additional 20 mL of saline solution and 20 mL of deionized water. The AuNW network was dried for 5 min @ 81 °C and stored for min 2 days before transfer to the elastomer film.

#### Sample Fabrication

Stretchable electronic samples were fabricated by embedding the AuNW network in five elastomers. The detailed fabrication process for each elastomer composite is explained in the Supporting Information. The general process is as follows. Embedding of the AuNWs in elastomers is achieved by placing the filter membrane with the deposited AuNW network upside down onto an 80 μm thick spin-coated layer of the respective elastomer in viscous form. The viscous elastomer partially infiltrates the AuNW network in this step. The elastomer with the filter membrane on top was cured/dried on a hot plate at ∼80 °C with 1 kg placed on the back of the filter membrane (low pressure to ensure conformal contact). The filter membrane was peeled off, leaving the AuNWs partially embedded on the elastomer film. The NWs were further embedded by spin coating of additional layers of elastomer to a total thickness of ∼160 μm while preventing the coverage of electrodes on both sides of the sample for contacting. The stretchable composite was cured/dried for at least 12 h at ∼80 °C and stored for an additional 48 h before any tests were conducted. Individual samples were cut out manually with a scalpel from the test films and peeled from the low adhesion substrate (trichloro(1H,1H,2H,2H-perfluorooctyl)silane-coated borosilicate glass wafer), and each sample’s exact width and thickness was determined with a microscope. Extra care was taken to ensure similar processing parameters for all elastomer composites. Pure elastomer samples were fabricated with the same processing steps but without the NW film.

### Mechanical Testing

#### Tensile Testing

Tensile tests were performed horizontally on a motorized linear stage (X-LSQ300A-E01, Zaber) equipped with a force gauge (Mark-10 M5-2, 10 N range) and controlled with a custom LabVIEW program. Force spike artifacts occurring due to the movement of the linear stage were removed from the data. Tensile tests were done on 2 mm wide rectangular samples, clamped 15 mm in between two rubber clamps and stretched at a velocity of 0.5 mm/s (3.3%/s). The samples showed a small initial slack of 1–2% defined by the strain at the initial force gauge signal, which was considered to determine tensile set values but not for any other calculations. All stress measurements are stated as engineering stress, not taking into account the deformation of the sample.

#### Pull to Failure

Three samples of each composite and pure elastomer were stretched until rupture. The modulus at 100% strain was extracted from the stress value at 100% (Table 1), and Young’s modulus (initial stress slope) was extracted by fitting the linear stress slope up to 10% strain (Figure S5). Filler stiffening due to AuNW embedding was calculated by dividing the mean value with AuNWs by the pure elastomer value.

#### Cyclic Loading with Increasing Amplitude

Three samples of each composite and pure elastomer were stretched consecutively to incrementally higher strains (cyclic loading to 10, 20, 50, 100, 200, 300, and 500%). The tensile set was calculated according to De and Gaymans^56^ for each consecutive stress cycle while subtracting the small initial slack of 1–2% common to all samples (defined by the strain at the initial force gauge signal).

#### Cyclic Loading for Stress Softening

One composite sample each was stretched for 100 cycles consecutively to 100% strain. To quantify the cycle dependency of deformation, the area under the loading curve (loading energy) was calculated for cycles 2, 5, 10, 20, 50, 80, and 100 and divided by the value of cycle 2 to exclude the effect of the initial cycle amplitude (Figure 5f).

#### Stress Relaxation

Each pure elastomer sample was stretched to 200% and 500% and held for 30 min while measuring the force. Stress values were normalized to initial maximal stress to get relaxation curves.

### Resistance-Strain Cycling

Electromechanical tests were performed horizontally on a motorized linear stage (X-LSQ300A-E01, Zaber) equipped with a four-probe setup connected to a Keithley 2701 digital multimeter and controlled with a custom LabVIEW program. The samples were taped and clamped slightly into the insulated part to avoid localized damage at the more sensitive contact pads (Figure S2). Three rectangular composite samples of 1 mm width were clamped on a distance of 16 mm and stretched at 2 mm/s (12.5%/s) for 1000 cycles to 20, 50, and 100% with a 1 s resting time between each cycle. The resistance was divided by the sample’s aspect ratio to extract their sheet resistance.

### Backlight Microscope Images

Backlight images with controlled settings were taken of AuNW–elastomer composites initially and after strain cycling for 1000 cycles. Images were taken at the respective cycling strain to expose crack formation during stretching (Figures 3a and S1). The 2-dimensional fast Fourier transform (FFT) of the images was calculated with Matlab (Mathworks) to highlight the periodicity of cracks in the backlight images (Figure 3a, left insets), while the crack directionality was extracted with the “directionality” plugin in Fiji (ImageJ), which bins the radial distribution of the FFT components (Figure 3a, right insets).

### Elastomer–Gold Interface Characterization

#### Sample Fabrication for Interface Studies

A 5 nm thick chromium and 25 nm thick gold layer was evaporated on a glass slide at a 2 Å/s evaporation rate. The resulting gold slides were stored 2 days in an acidic poly(vinylpyrrolidone) (PVP) solution and subsequently thoroughly rinsed with saline solution and deionized water to achieve a similar surface to the AuNWs, which also exhibit the remaining PVP layer even after cleaning. The resulting gold slides (AuPVP) were stored for 2 days, and a thick film of elastomer was spin-coated on top and reinforced with a cleanroom tissue.

#### Surface Free-Energy Measurements

To measure the surface free energy of the different elastomers and AuPVP, a Krüss Mobile Surface Analyzer (MSA) was used. The MSA dispensed droplets of two liquids of different polarities onto the test surface: polar water and nonpolar diiodomethane. From a built-in camera feed, the system measured all contact angles for the droplets against the surface and the surrounding air and subsequently calculated the surface free energy for the surface as well as the polar energy component via the Krüss Advance software (Figures 3b and S2).

#### Peeling Tests

Peeling tests to measure adhesion force between different elastomers and AuPVP were carried out using a Universal Testing Machine ZPM with a 100 N head setup. Force meter output was processed with THSSD-2018 software. The elastomer strips measured 19 mm width and 45 mm scanned length. The peeling was carried out at a speed of 30 mm/min for all elastomers except WPU, which was peeled at a speed of 10 mm/min. The table to which the sample was attached moved along as the elastomer was peeled upward in order to maintain a constant 90° peeling angle.

### Scanning Electron Microscopy

AuNW–elastomer composite samples were submerged in liquid N_2_ and cracked to obtain an undistorted cross section. Samples for Figure 3c were cycled 200× to 100% strain, and after cracking in liquid N_2_, they were fixed at ∼50% strain on Kapton tape prior to imaging. Scanning electron microscopy (SEM) images were taken with a Zeiss Sigma 500 Gemini using a 1–2 kV acceleration voltage with an In-Lens detector.

### X-ray Tomography

AuNW–elastomer composite samples were cycled for 1000 cycles to 100% strain and consecutively fixed at 100% strain in between two Kapton tapes. The samples were scanned using the Zeiss Versa Xradia 520 at Stockholm University Brain Imaging Centre (SUBIC). During the scan, the X-ray source was set to a voltage of 100 kV and a power of 9 W. The 20× objective was utilized, resulting in effective pixel sizes between 0.36 and 0.49 μm. These sizes depended on the distances between the detector, sample, and objective for the scans, which were optimized for each sample based on its width. Each tomography scan comprised 1601 projections over 182°. The exposure time for each projection ranged from 15 to 30 s for different samples, depending on the photon counts affected by the distances.

To segment 3D AuNW images, we first cropped and aligned the images in Dragonfly Version 2022.2 for [Windows] (Comet Technologies Canada Inc., Montreal, Canada; software available at https://www.theobjects.com/dragonfly) to 400 × 400 × 50 μm^3^ volume with the AuNW layer positioned at the center. Subsequently, we analyzed the pure elastomer matrix, having the same volume size and orientation, using MATLAB R2022a for segmentation. This step was essential due to variations in voxel size, intensity, and distribution. For the FVMQ AuNW sample, the cutoff was set at mean + 4 standard deviations from the pure FVMQ elastomer data to ensure the removal of the pure elastomer contribution within the volume. The cutoff for other samples was set to achieve the same volume of AuNWs, accurately representing the actual samples for fair comparison. This process involved exporting the voxel distribution from Dragonfly and analyzing the data using a MATLAB R2022a code.

## Supporting Information Available

Details on sample fabrication, SEM images of AuNW layer thicknesses, schematics and photo of clamping, backlight microscopy images, surface free-energy measurements, Young’s modulus of the investigated elastomers, sheet resistance before and after peeling, resistance-strain measurements, cyclic strain at different rates, X-ray tomography images, and resistance measurements during sample relaxation (PDF)
