# Supplementary material for: Exploring the Elastomer Influence on the Electromechanical Performance of Stretchable Conductors
Source: ACS Appl Mater Interfaces. 2024 Jul 9;16(29):38365–76. doi: 10.1021/acsami.4c03080 (PMC11284748; doi:10.1021/acsami.4c03080)
Supplement: Supplementary file 1 — am4c03080_si_001.pdf [file am4c03080_si_001.pdf]

Supporting Information

Exploring the elastomer influence on the electro-mechanical performance of stretchable conductors

Samuel Lienemann[a], Ulrika Boda[a,b], Mohsen Mohammadi,[a] Tunhe Zhou,[c] Ioannis Petsagkourakis[b], Nara Kim[a] and Klas Tybrandt[a,]*

[a] Laboratory of Organic Electronics, Department of Science and Technology, Linköping University, 601 74 Norrköping, Sweden.

[b] Bio- and organic electronics unit, RISE Research Institutes of Sweden, 602 33 Norrköping, Sweden

[c] Stockholm University Brain Imaging Centre (SUBIC), Stockholm University, 106 91 Stockholm, Sweden

[*] Email: klas.tybrandt@liu.se

Details on sample fabrication

The substrate was in all cases a silanized (Trichloro(1H,1H,2H,2H- perfluorooctyl)silane) borosilicate glass wafer. The gold nanowires (AuNWs) are filtered onto a PVDF membrane as described in the main manuscript. Heat was applied on a hotplate with smooth surface and well controlled heat conduction. Contact protections were made from 25 µm thick polyethylene-naphthalate (PEN) except for WPU.

PDMS:

1. mix Sylgard 184 base with curing agent 10:1 in planetary mixer (Thinky)
2. spincoat PDMS at 850 rpm for 30 s
3. semicure at 70 °C for 4-7 min until PDMS feels solid but sticky.
4. roll on membrane with AuNWs
5. place foam and 1kg on back to ensure conformal contact
6. cure 10 min at 70 °C
7. immerse in deionized water and peel filter membrane off, and dry at room temperature
8. infiltrate with heptane:PDMS (20:1 w/w), spincoat immediately to prevent swelling at 6000 rpm for 60 s, subsequently cure 10 min at 70 °C
9. encapsulate at 1000 rpm for 30 s with PEN foil as contact protection
10. peel PEN foil, cure over night at 70 °C

FVMQ:

1. Nusil MED-5440, 1:1 mixture from dispenser. Spread generously on wafer, prevent air entrapment.

2. spin coat at 1000 rpm for 30 s (spread) and 9000 rpm for 120 s
3. semi cure at 75 °C for 2:30 min (still viscous)
4. Roll on membrane with AuNWs softly (prevent sliding), place foam and 1kg on back to ensure conformal contact.
5. cure 15 min at 75 °C
6. immerse in deionized water and peel filter membrane off and dry at room temperature
11. infiltrate with butanone:nusil(16:1 w/w), spincoat immediately to prevent swelling at 6000 rpm for 60 s, subsequently cure 5 min at 75 °C
7. encapsulate at 1000 rpm for 30s plus 9000 rpm for 120s with PEN foil as contact protection
8. peel PEN foil, cure over night at 75 °C

<u>TPU</u>:

1. dissolve Elastollan® Soft 35 A 12 (500mg/mL) in dimethylformamide over night at 80 °C
2. spin coat at 800 rpm 30 s (plus 2 s 1500 rpm), semi-dry 2 min at 75 °C, spin additional layer at 1200 rpm 30 s
3. Semi-dry 7 min at 75 °C
4. roll on membrane with AuNWs and place foam and 1kg on back to ensure conformal contact.
5. dry 5 min at 75 °C, remove weight, dry another 5 min at 75 °C, peel filter membrane.
6. encapsulate at 600 rpm for 30 s, with PEN foil as contact protection
7. peel PEN foil, bake over night at 75 °C

<u>WPU</u>:

1. spin coat Alberdingk® U 4101 VP from solution 5 layers at 400 rpm for 30 s (plus 2 s 1000 rpm), dry 2 min at 75 °C in between each spin coating
2. semidry the 5th layer at room temperature for 4 min 30s
3. roll on membrane with AuNWs and place foam and 1kg on back to ensure conformal contact
4. dry at 75 °C for 4 min. and additional 5 min after removing the weight
5. immerse in deionized water, peel filter membrane off and dry at room temperature
6. cover contacts with 50 μm thick PDMS during encapsulation, spin 3 layers at 400 rpm for 30 s (plus 2 s 1000 rpm) dry 2 min at 75 °C in between each spin coating and place new PDMS foil
7. bake over night at 75 °C

<u>SEBS</u>:

1. dissolve Tuftec H1052 (400mg/mL) in Toluene over night at 90 °C
2. spin coat at 500 rpm for 1 min (plus 2.5 s 1500 rpm)
3. semi-dry 1 min at 50 °C
4. roll on membrane with AuNWs and place foam and 1kg on back to ensure conformal contact
5. dry 10 min at 50 °C, remove weight, dry another 5 min at 75 °C, peel filter membrane
6. immerse in deionized water, peel filter membrane off and dry at room temperature
7. spin coat at 1500 rpm for 30s with PEN foil as contact protection

8. peel PEN foil, dry 10 min at 50 °C then bake over night at 75 °C

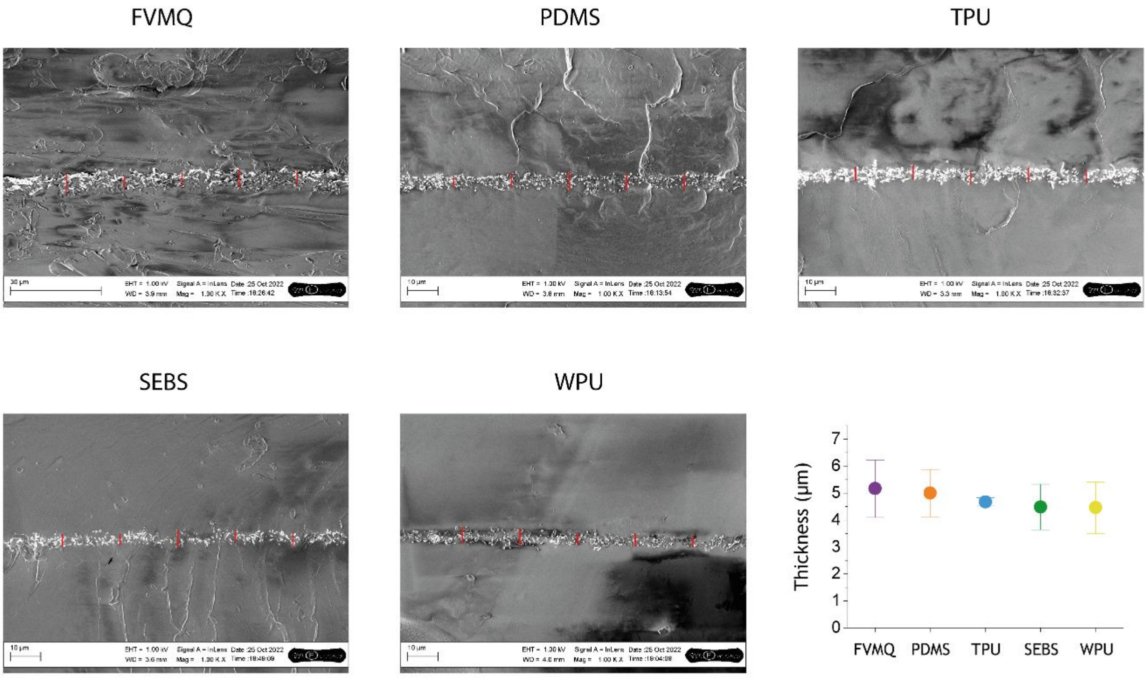

Figure S1. AuNW-layer thickness for the different elastomer samples.

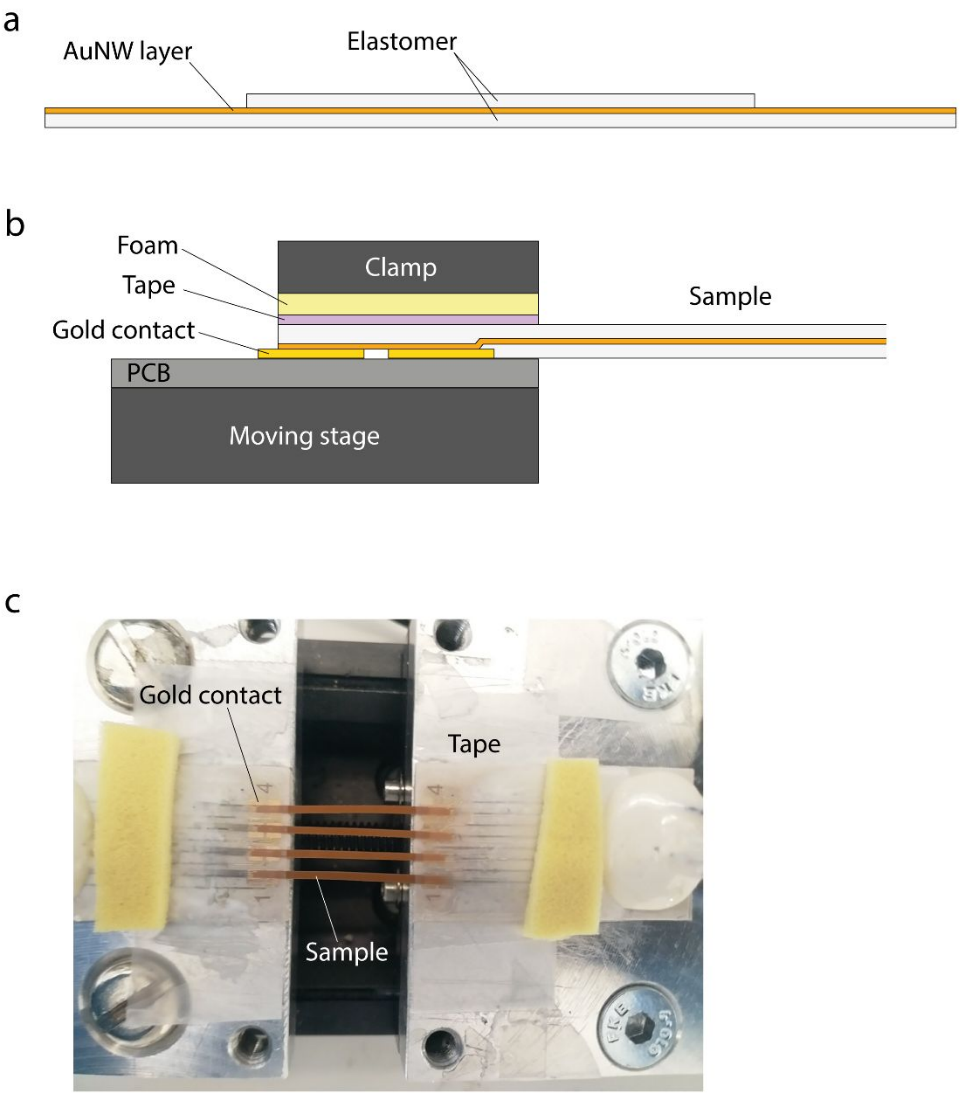

Figure S2. a) Sample structure. b) Schematic of contacting and clamping the sample. The tape in combination with the mechanical clamping ensures stable electrical contact and stable mechanical anchoring. c) Photograph of the sample mounting prior to mechanical clamping.

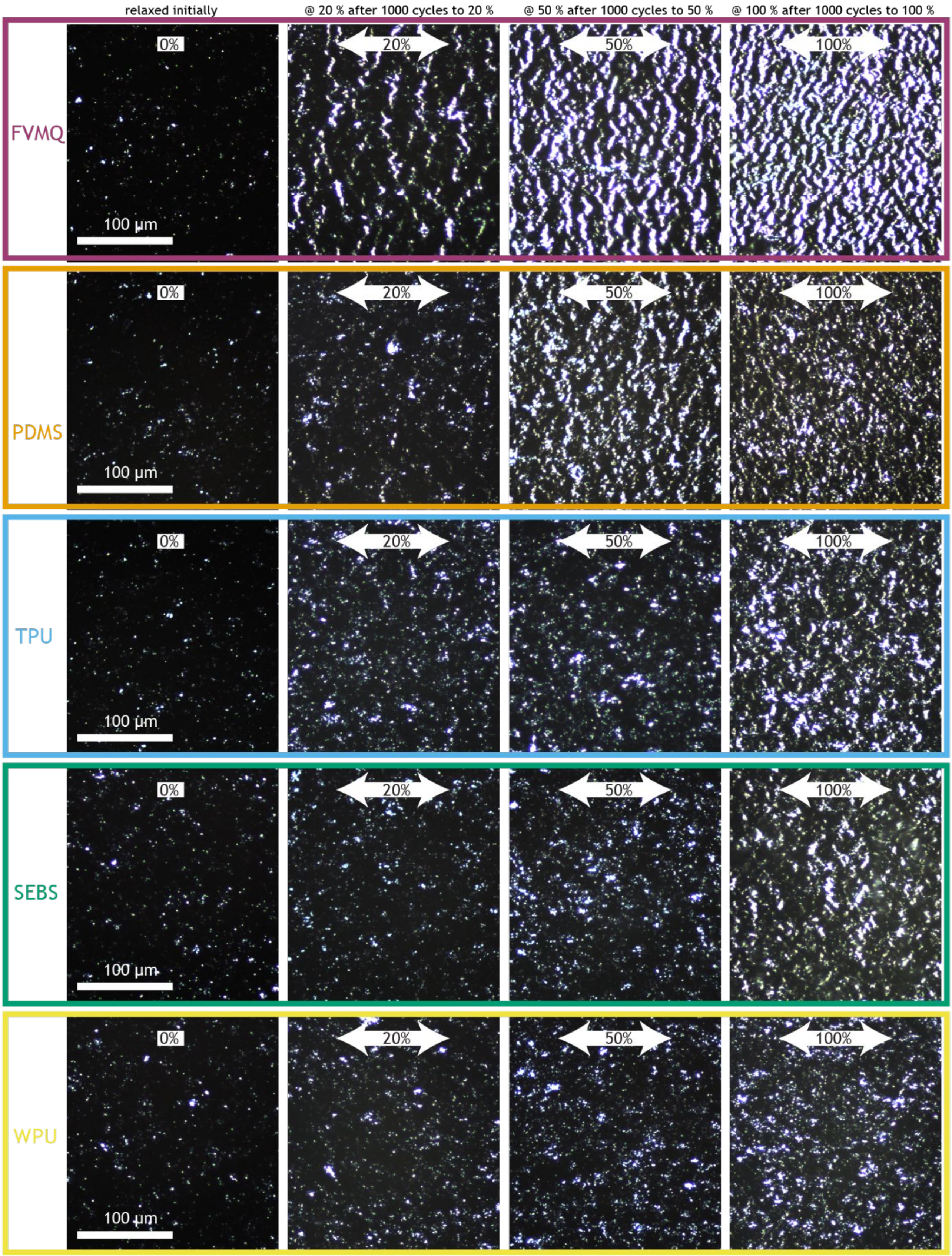

Figure S3. *Representative backlight microscope pictures of AuNW-elastomer composites initial, and after 1000 cycles to 20, 50 and 100 % strain, respectively. Image taken at the respective strain the sample was subjected to during strain cycling. scale bar: 100 µm for all images.*

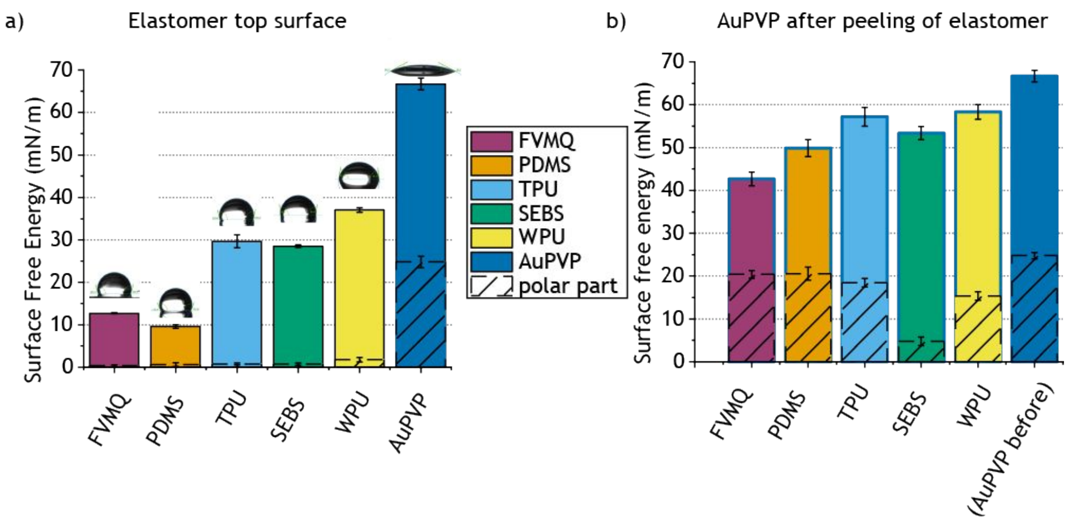

***Figure S4*. *a)* Surface free energy of pure elastomer with exemplary water droplets depicted above the respective value. *b)* Surface free energy of the gold slides from the peeling tests after peeling of the elastomer.**

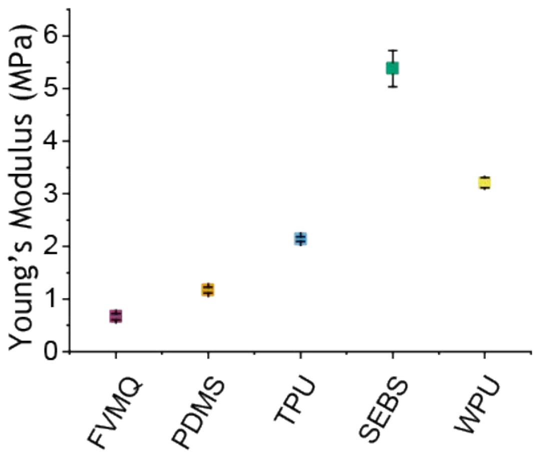

Figure S5. *Young's modulus of the investigated elastomers extracted from the linear fit of the initial stress slope to 10 % strain.*

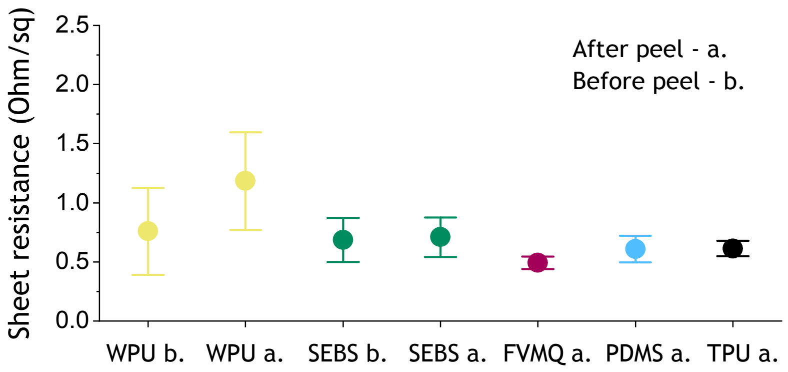

Figure S6. Sheet resistance of WPU and SEBS samples before and after peeling compared to peeled FVMQ, PDMS and TPU samples (2-probe measurements).

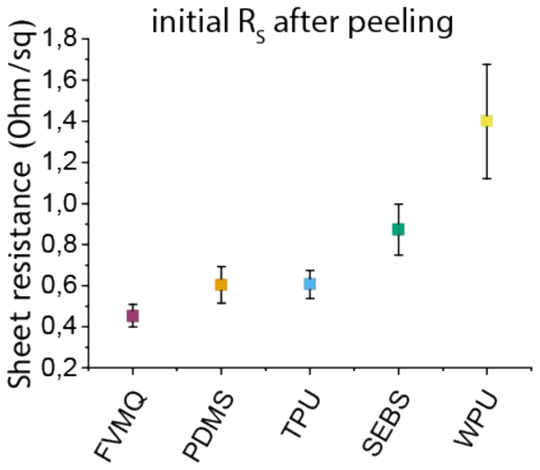

Figure S7*. Sheet resistance of the samples after peeling measured on 4-probe-set-up before strain cycling.*

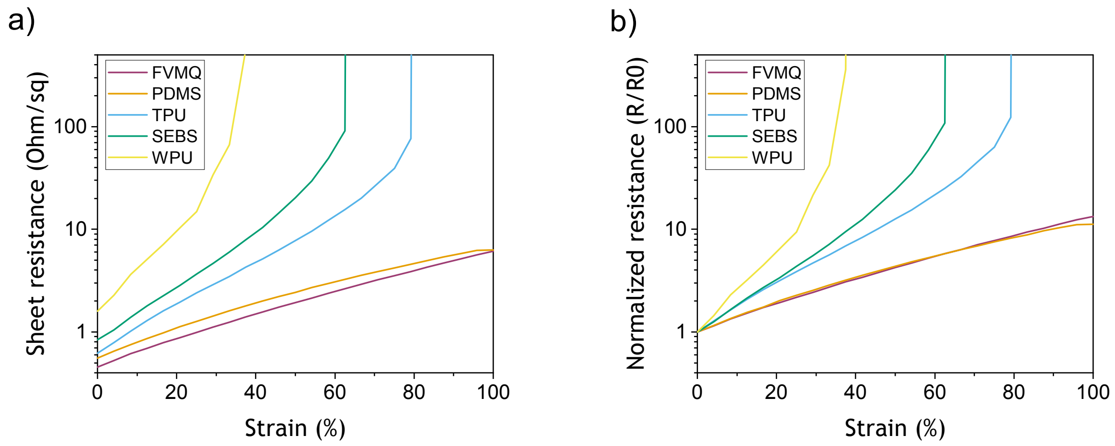

Figure S8. a) Sheet resistance for the various elastomer samples for a single stretch to 100% strain. b) Normalized resistance for the various elastomer samples for a single stretch to 100% strain.

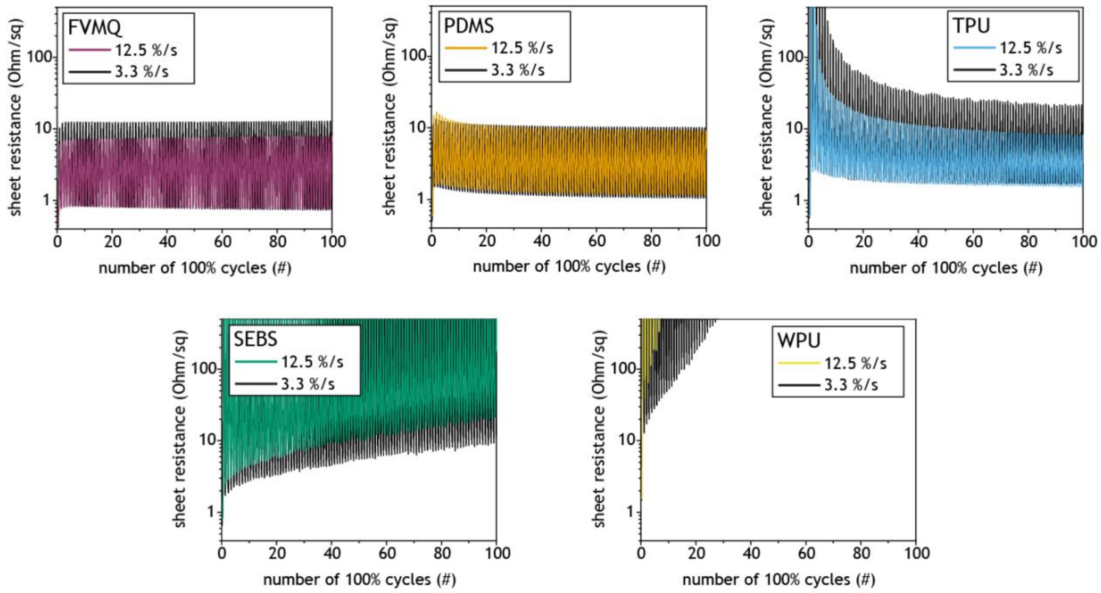

Figure S9. *Conductor resistance during strain cycling to 100% strain at two different strain rates for the AuNWs in five elastomers. Colored lines are the mean values of the samples stretched at 12.5 %/s (same values as in Figure 2c) and the black line shows data for a slower strain rate (3.3 %/s, n=1, 2-probe set-up). The resistance-cycle trend is similar for the different strain rates for all conductors.*

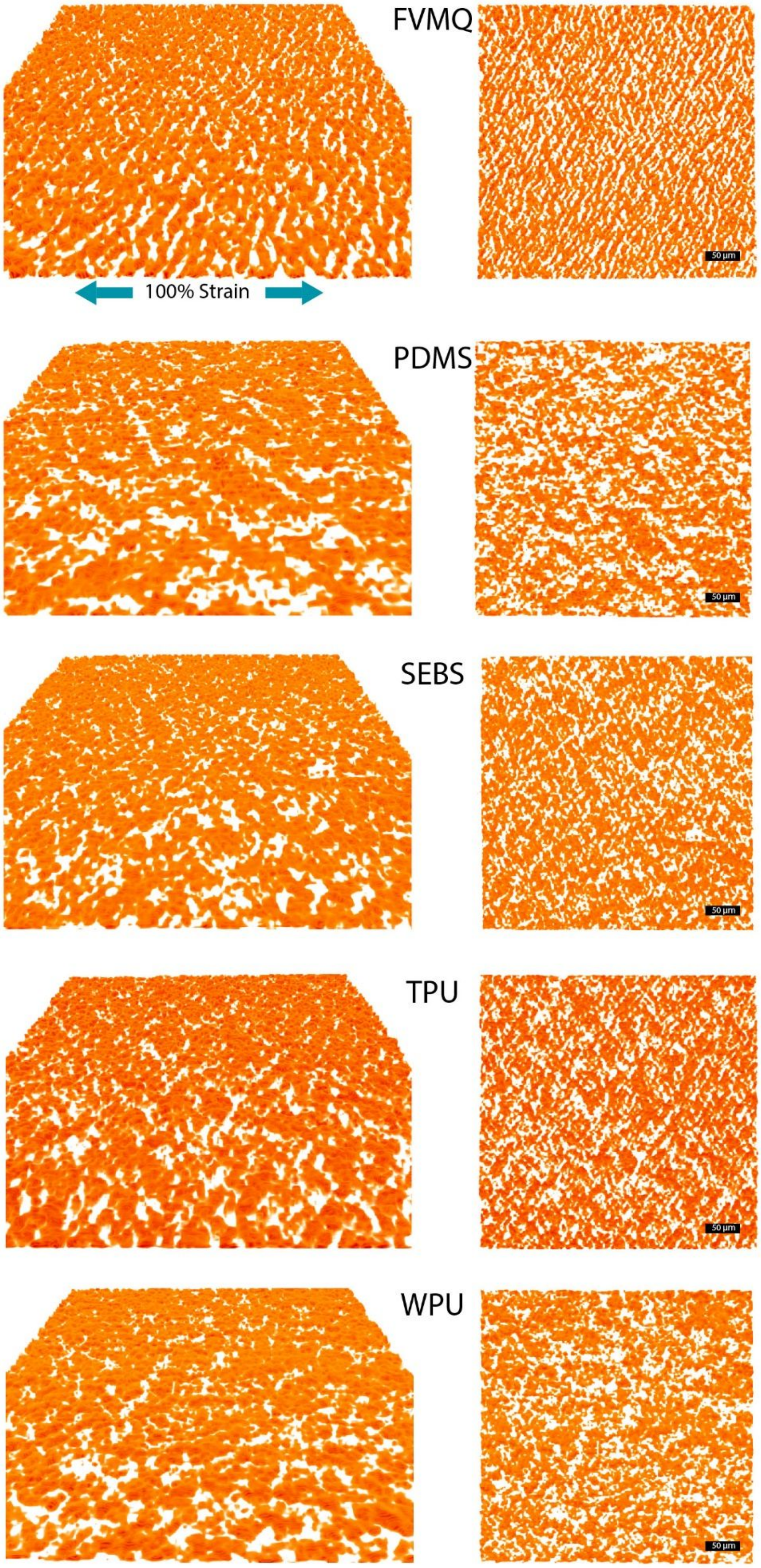

Figure S10. X-ray tomography images from two different angles at 100 % strain for strain cycled conductors.

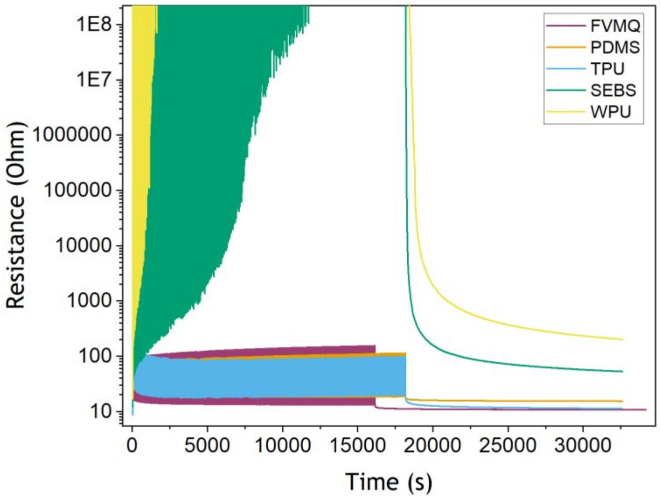

Figure S11. Strain cycling (100% strain, 1000 cycles) followed by monitoring of the resistance with time for relaxed samples. The SEBS and WPU samples go from non-conductive at the end of the cycling to moderately conductive during the relaxation.
